# Late effects and treatment related morbidity associated with treatment of neuroblastoma patients in a tertiary paediatric centre

**DOI:** 10.1002/cnr2.1738

**Published:** 2022-10-21

**Authors:** Veronica Yeung, Melissa Gabriel, Bhavna D. Padhye

**Affiliations:** ^1^ ProCan®, Children’s Medical Research Institute, Faculty of Medicine and Health The University of Sydney Westmead NSW Australia; ^2^ Cancer Centre for Children The Children's Hospital Westmead Westmead NSW Australia

**Keywords:** childhood cancer, late effects, long‐term toxicity, neuroblastoma, survivorship

## Abstract

**Background:**

Survival of neuroblastoma patients has improved over recent decades, but chronic health issues and treatment related late effects cause significant morbidity in survivors.

**Aims:**

We aimed to describe late effects and long‐term toxicity in neuroblastoma patients treated at a tertiary, paediatric institution in Australia.

**Methods & Results:**

Patients with neuroblastoma treated primarily at The Children's hospital at Westmead were eligible for inclusion. Retrospective analysis of 65 (45 with high‐risk and 20 with non‐high‐risk disease) neuroblastoma patients were performed via medical record review. Approximately 60% of patients were >5 years from diagnosis and termed the “full effects cohort” who had a range of medical and psychosocial late effects analysed through descriptive means. The remaining 26 patients who had not yet reached 5 years post treatment had audiometry analysis only. Of the 65 patients, 72% were alive at last follow‐up. The median length of follow‐up was 7 years from diagnosis amongst survivors. Therapy was according to contemporary protocols for neuroblastoma and ranged from standard cytotoxic therapies to intensive multimodal regimens and/or experimental therapy depending on risk group/relapse status. Of the 39 full effects cohort, 85% suffered from at least one late effect. Late effects were common in the endocrine, dental and audiometry domains with 38%, 49% and 72% of patients affected in these areas, respectively. Neuro‐cognitive domains were also notably affected with 46% of patients suffering a deficit. Two thirds of survivors were disease free at last follow‐up.

**Conclusion:**

Survivors of high‐risk neuroblastoma suffer from a range of chronic illnesses, which lead to morbidity and affect quality of life of survivors.

## 1 INTRODUCTION

Neuroblastoma is the most common extracranial solid tumour in children.^1^, ^2^ It accounts for approximately, 6%^2^ of childhood malignancies and survivors develop late effects across multiple domains. Survival rates for neuroblastoma vary significantly based on risk group.^3^ Therapy for non‐high‐risk (NHR) neuroblastoma has evolved with de‐escalation over the years, allowing for high rates of survival with reduced long‐term morbidity, whilst treatment for high‐risk (HR) neuroblastoma has intensified, which has also increased risk of long‐term side‐effects.

Risk stratification in neuroblastoma is based on a combination of biological and histological features, along with patient related characteristics such as age.^4^ Patients in the HR group are treated with a combination of conventional chemotherapy,^5^ surgery, high‐dose chemotherapy followed by autologous stem cell transplant (ASCT), radiotherapy, immunotherapy with anti‐GD‐2 antibody and isotretinoin. Conventional induction chemotherapy^5^ includes cisplatin, vincristine, etoposide, topotecan, doxorubicin and cyclophosphamide, which contribute to late toxicity. The spectrum of late effects varies, with significant impacts on hearing and dental outcomes, as well as endocrine consequences, particularly affecting growth and thyroid function.^2^, ^6^ Additional late effects also occur in cardiac, renal, respiratory and neuro‐cognitive domains including behavioural, speech and learning deficits, affecting overall mental health outcomes.^2^, ^6^, ^7^

A large cohort analysis of neuroblastoma survivors in North America^8^ has begun to define the range of health consequences suffered after Children's Oncology Group (COG) based treatment. The ALTE15N2 COG non‐therapeutic study^8^ focuses on assessing the long‐term side‐effects of neuroblastoma treatment from patients enrolled on the COG Neuroblastoma Biology study (ANBL00B1) and results suggest an increasing number of survivors are facing a range of toxicity profiles. Australian data on neuroblastoma survivorship is limited to a single institution^9^ analysing the long‐term health outcomes of HR Neuroblastoma patients treated with ASCT, demonstrating that 26 of 40 patients developed at least one treatment related late effect, which included renal, audiometry, endocrine/bone metabolism disorders and secondary malignancies. The purpose of our study is to review and characterize data of neuroblastoma patients treated in a tertiary paediatric referral centre in Australia, the Children's Hospital at Westmead (CHW), focusing on late effects including subsequent neoplasms and chronic health challenges. Additionally, this study will compare the results of CHW to international cohort analyses, including studies involving the Childhood Cancer Survivor Study with similar research objectives.

## 2 METHODS

At CHW, patients are followed up for 5 years by their oncologist after completion of therapy. They are then referred to a multidisciplinary (MDT) long‐term follow‐up clinic (LTFU) for ongoing late effects monitoring, and management.

### 2.1 Data

**FIGURE 1:**
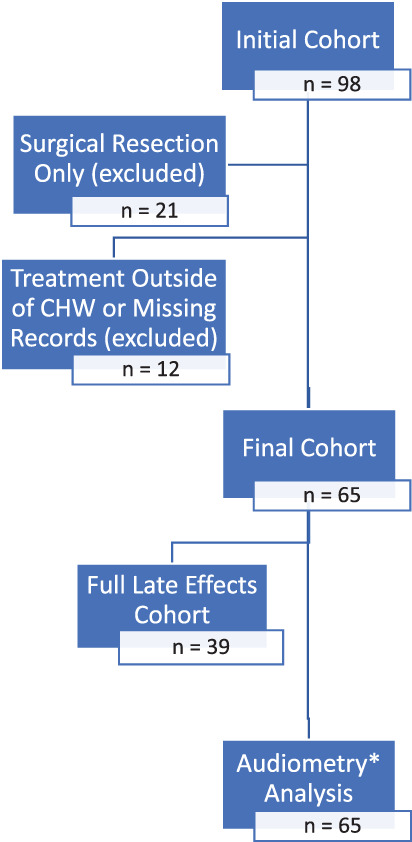
Exclusion criteria to derive final cohort for analysis and breakdown into subgroups. “*” 26 patients did not have full late effects analysis as they had not reached 5 years post treatment, but all patients had audiometry analysis.

Data were collected through retrospective review of electronic medical records of children diagnosed with neuroblastoma from 1 January 2009 to 31 December 2019 treated primarily at CHW, where treatment included chemotherapy as a baseline. Cases initially included had a histological diagnosis of neuroblastoma, ganglioneuroblastoma or ganglioneuroma totalling 98 patients. Of these 98 patients, 21 were excluded because chemotherapy was not part of treatment (surgical resection alone), and 12 were excluded either because primary treatment was given outside of CHW, or medical records were absent. A total of 65 patients were included in the final analysis after the above exclusions (Figure 1).

Of these 65 patients, analysis was further subdivided into two groups. The “full late effects cohort” included all patients diagnosed before 2015; they had a full medical record review inclusive of major late effects including endocrine, cardiac, dental, audiometric impairment, respiratory, neurobehavioral deficits and subsequent malignant neoplasms (SMN); patients diagnosed from 2015 and onwards were subject to audiometry record review only. Decision to separate the cohort into two groups was to allow for ongoing review of late effects in patients who would not have reached LTFU by 5 years post treatment. Review of records occurred in May 2021. There were 39 patients in the full late effects cohort and 65 in the audiometry analysis group (Figure 1). Audiometric impairment was defined by Chang^10^ grades in this study as the standard of measure rather than the common terminology criteria for adverse events (CTCAE)^11^ (Tables S1 and S2). Data were de‐identified and given a unique identifier once collated into a master dataset for descriptive analysis.

### 2.2 Analysis

We analysed 65 patients in total diagnosed over a 10‐year period. The timepoint when medical record review occurred was defined as follow‐up. Age at diagnosis, age at follow‐up, and number of years from diagnosis to record review, defined as length of follow‐up were recorded. Descriptive statistics were derived for these respective categories. Both clinical and therapy‐based risk groups were recorded, but patients were analysed according to therapy received rather than clinical risk group and used to divide the late effects for analysis. Late effects analysed included endocrine, cardio‐pulmonary, dental, audiometry, neuro‐cognitive and SMNs. Presence of late effects were included if there was confirmation of a particular late effect in the medical records. For instance, endocrine effects were recorded if an endocrinologist or an oncologist in the LTFU clinic diagnosed the condition. Audiometry effects were recorded based on audiogram results, cardio‐pulmonary effects were recorded based on testing through echocardiograms/electrocardiograms or pulmonary function tests. Dental effects were recorded based on findings from dental examination, and neuro‐cognitive effects were based on issues identified by the LTFU clinic, or paediatric specialists such as a general paediatrician, or neurologist. Baseline characteristics of patients were recorded and analysed. Patients were divided into therapy‐based risk groups; HR patients received aggressive therapy that included a combination of HR chemotherapy with one or more treatment modality such as surgery, autologous transplant, and so forth, whilst NHR patients must have received intermediate risk chemotherapy plus or minus another treatment modality. Summary statistics were used to describe the cohorts. Continuous variables were summarized using median values and categorical variables using proportions. Statistical comparisons were performed using statistical software (*Jamovi*, version 1.6) and a two‐sided Fisher's exact test (FET) was used to compare the HR and NHR therapy groups in each late effect domain, except for the cardio‐pulmonary domain as the overall numbers were small. Survival analyses were performed using the Kaplan–Meier method and compared using the log‐rank test. Microsoft Excel was used to record and collate our data.

Descriptive survivorship data of disease status, morbidity and mortality were also analysed and divided based on therapy‐based risk groups. Comparisons of HR versus NHR survivors in each late effect domain only included patients from the “full late effects cohort” who were still alive at follow‐up, except for audiometry where all 65 patients were assessed.

## 3 RESULTS

### 3.1 Demographic data

Baseline characteristics of the 65 patients included in this study are given in Table 1. Just over half 36/65 (55%) were females. More than two thirds of patients 45/65 (69%), were HR and 20/65 (31%) were NHR. The median age at diagnosis was 2.1 years (interquartile range, IQR 1.1–3.4 years).

**TABLE 1:** Demographics and baseline characteristics of neuroblastoma patients treated at CHW from 1 January 2009 to 31 December 2019 (*n* = 65) based on high‐risk and non‐high risk therapy groups

Characteristics	High‐risk	Non‐high risk	Total
Total no.	45	20	65
Gender no. (%)			
Female	21 (47)	15 (75)	36 (55)
Male	24 (53)	5 (25)	29 (45)
Median age at diagnosis in years (IQR)	2.6 (1.8–3.8)	0.7 (0.5–1.4)	2.1 (1.1–3.4)
Age at diagnosis			
0–18 months no. (%)	8 (18)	15 (75)	23 (35)
≥18 months no. (%)	37 (82)	5 (25)	42 (65)
Histology			
Favourable no.	7	13	20
Unfavourable no.	28	4	32
Ganglioneuroblastoma no.	1	0	1
NOS no.	9	3	12
NMYC status			
Amplified no. (%)	20 (44)	0 (0)	20 (31)
Not amplified no. (%)	25 (56)	20 (100)	45 (69)
Survival			
Survivors no. (%)	28 (62)	19 (95)	47 (72)
Median age at follow‐up of survivors in years (IQR)	11.2 (7.6–13.7)	7.5 (4.2–9.3)	9.1 (6.3–12.6)
Median length of follow‐up of survivors from diagnosis in years (IQR)	9.1 (4.7–10.6)	6.4 (2.8–7.8)	7.0 (3.4–10.0)

Abbreviations: CHW, The Children's Hospital at Westmead; HR, high‐risk; IQR, interquartile range; NHR, non‐high risk; NOS, not otherwise specified.

A total of 47 out of 65 patients (72%) were alive at follow‐up, 28 in the HR group and 19 in the NHR group (Table 1). The median age at follow‐up of all survivors was 9.1 years (IQR 6.3–12.6). The median length of follow‐up from diagnosis amongs survivors was 7.0 years (IQR 3.4–10.0). Median age of follow‐up in the HR survivors was 11.2 years (IQR 7.6–13.7) and 7.5 years for NHR survivors (IQR 4.2–9.3).

Demographic details, histology and NMYC status of all 65 patients are described in Table 1.

### 3.2 Treatment received

Treatment received overall for all patients is stratified by risk group and shown in Table 2. HR patients who received the full course of contemporary therapy including HR chemotherapy, surgery, ASCT, radiotherapy, immunotherapy and isotretinoin totalled 33/45 (73%). The other 12/45 (27%) of HR patients received some combination of these therapies (Table 2). Looking at patients by clinical risk groups, 44 HR patients underwent HR therapy, but 1 NHR patient also undertook HR therapy inclusive of surgery, chemotherapy and ASCT secondary to poor response to standard therapy. Of the 20 NHR patients, 11 received a combination of surgery and chemotherapy as the majority treatment and 6 received chemotherapy alone. Additional therapy information was also determined; 11/45 HR patients received metaiodobenzylguanidine (MIBG), experimental therapies (based on molecular analysis), or a combination of the two as part of their total treatment due to refractory disease or relapse.

**TABLE 2:** Treatment and therapies received stratified by risk group in all patients (*n* = 65).

	Therapy received		
	High‐risk (45)	Non‐high risk (20)	Total (65)
Clinical risk group no.			
High‐risk	44	1	45
Non‐high risk^a^	1	19	20
Overall treatment specifics no.			
Chemotherapy only	0	6	6
Surgery and chemotherapy	3	11	14
Surgery, chemotherapy, ASCT	1	0	1
Surgery, chemotherapy, ASCT, RA	1	0	1
Surgery, chemotherapy, ASCT, radiation	2	0	2
Surgery, chemotherapy, ASCT, radiation, RA	1	0	1
Surgery, chemotherapy, immunotherapy	2	0	2
Surgery, chemotherapy, RA	0	2	2
Surgery, chemotherapy, radiation	1	0	1
Surgery, chemotherapy, radiation, immunotherapy, RA	1	1	2
Surgery, chemotherapy, ASCT, radiation, immunotherapy, RA	33	0	33
Additional therapy to above no.	11	1	12
MIBG therapy alone	6	0	6
Lutate therapy	0	1	1
Experimental therapy alone	3	0	3
MIBG and experimental therapy	2	0	2

Abbreviations: ASCT, autologous stem cell transplant; HR, high risk; MIBG, metaiodobenzylguanidine; NHR, non‐high risk; RA, isotretinoin.

^a^ 1 NHR patient received individualized chemotherapy, surgery and ASCT secondary to poor response to therapy attributing the NHR patient receiving HR therapy.

Transplant details were recorded for all 65 patients including patients who were not transplanted (Table 3). Busulphan/melphalan was the most common conditioning regimen with 22 HR patients receiving this treatment. A total of 38 patients out of 65 underwent transplantation.

**TABLE 3:** Transplant details for all patients (*n* = 65).

	High risk (45)	Non‐high risk (20)	Total (65)	Survivors (47)
Single ASCT	33	0	33	24
Tandem ASCT	5	0	5	4
No ASCT	7	20	27	19
Conditioning regimen				
Busulphan/melphalan	22	0	22	15
Carboplatin/etoposide/melphalan	10	0	10	8
Melphalan only	1	0	1	1
Melphalan only (1st ASCT); Busulphan/cyclophosphamide (2nd ASCT)	1	0	1	1
Thiotepa/cyclophosphamide (1st ASCT); carboplatin/etoposide/melphalan (2nd ASCT)	4	0	4	3

Abbreviation: ASCT, autologous stem cell transplant.

### 3.3 Late effects

Overall make‐up of the 39 patients included 28 HR and 11 NHR patients. A total of 85% or 33/39 of the full late effects cohort had at least one late effect at follow‐up; 26/28 (93%) patients were of HR origin and 7/11 (64%) were NHR (*p* = .042, FET). Of the 6 with no late effects 2 had HR disease and 4 had NHR disease. The 2 patients with HR disease died secondary to disease relapse/progression at follow‐up at 2.5 years, and 10 months after diagnosis respectively.

### 3.4 Survival

**FIGURE 2:**
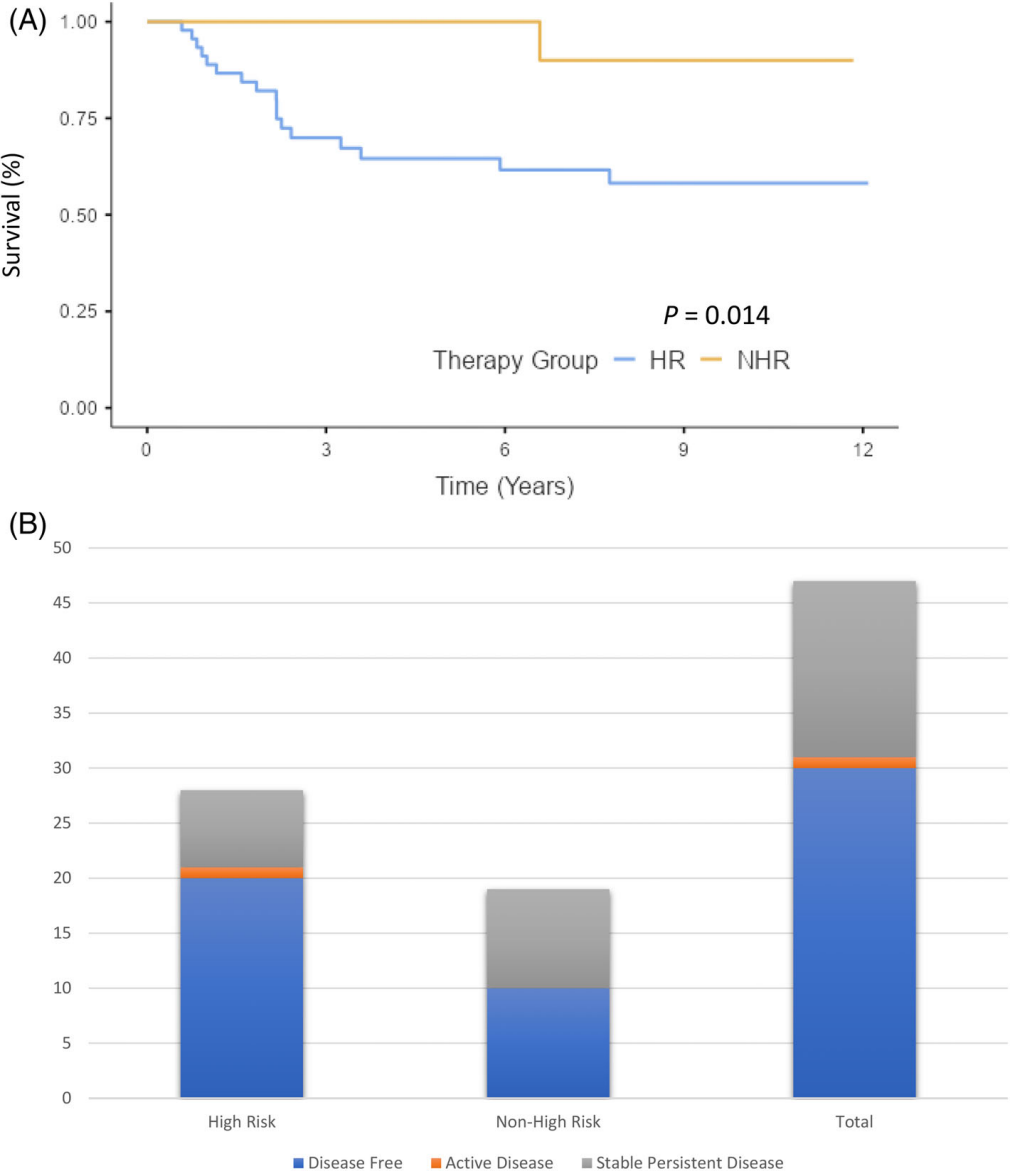
(A) Survival data for high‐risk (HR) and non‐high risk (NHR) patients in overall cohort *n* = 65. The *P* value is for the log‐rank test. (B) Disease free, active disease or stable persistent disease by risk group in survivors at last follow‐up, *n* = 47

Of the 65 full cohort patients, 47 patients were alive at follow‐up (72%); 28 were HR and 19 NHR (Figure 2A). A total of 30 out of the 47 live patients (64%) were disease free at follow‐up with 20 from the HR group and 10 from the NHR group. Stable persistent disease was found in 16/47 (34%) survivors with 7 HR and 9 NHR patients, respectively. Stable persistent disease was defined as abnormality on imaging, either persistent MIBG activity or on anatomical imaging. Figure 2B illustrates the total numbers of disease free, active disease, and stable persistent disease by risk group.

Of the 65 full cohort patients, 22 patients had either developed progressive disease or relapsed. A total of 18 out of the 65 patients were deceased; 17 were HR and 1 was NHR. The 1 NHR patient died of respiratory failure as a late effect 6.5 years post diagnosis. In the 17 HR patients, 16 deaths were attributed to progressive disease or relapse and 1 to respiratory failure as a late effect.

There were 27 survivors of the 39 patients in the late effects cohort at follow‐up; 17 were HR and 10 were NHR. All 17 of these HR patients (100%) experienced at least 1 late effect versus 6/10 (60%) of NHR patients (*p* = .012, FET).

### 3.5 Audiometry

Full cohort (*n* = 65) audiometry analysis was undertaken for all patients. Only 61 patients were evaluable with 4 patients demonstrating missing records. A total of 44 out of 61 (72%) patients were found to have some form of hearing impairment with sub‐analysis of grades as given in Table 4. A total of 41 out of 45 (91%) HR patients were hearing impaired versus 3 of 16 (19%) NHR patients (*p* = <.001, FET). The spectrum of hearing impairment ranged from Chang grade 1a to 4 with 10 patients falling into the unknown category when no grade was clearly documented. Of the survivors, 26/28 (93%) of the HR group were hearing impaired versus 2/19 NHR (11%) (*p* = <.001, FET), giving a total of 28/47 (60%) survivors with any form of hearing impairment (Table S3). Eight of the total survivor group had unknown hearing impairment grades.

**TABLE 4:** Breakdown of grades of audiometric impairment in the 44 hearing impaired patients.

Grade of audiometric impairment	High risk total 41 (out of 45 HR patients)	Non‐high risk total 3 (out of 16 NHR patients)	Overall total 44 (out of 61 evaluable patients)
Chang 1a	7	0	7
Chang 1b	3	0	3
Chang 2a	8	0	8
Chang 2b	1	0	1
Chang 3	10	0	10
Chang 4	2	0	2
Unknown	10	3	13

Abbreviations: HR, high risk; NHR, non‐high risk.

Management for hearing impairment ranged from hearing aids to radio frequency amplification systems with referral to Hearing Australia for follow‐up. Children who had shown signs of developmental/behavioural concerns, or who were at risk of developmental concerns were referred to a tertiary outpatient clinic for MDT management. MDT management included review by a paediatrician and referral for speech pathology among other non‐medical interventions such as hearing support teachers.

**FIGURE 3:**
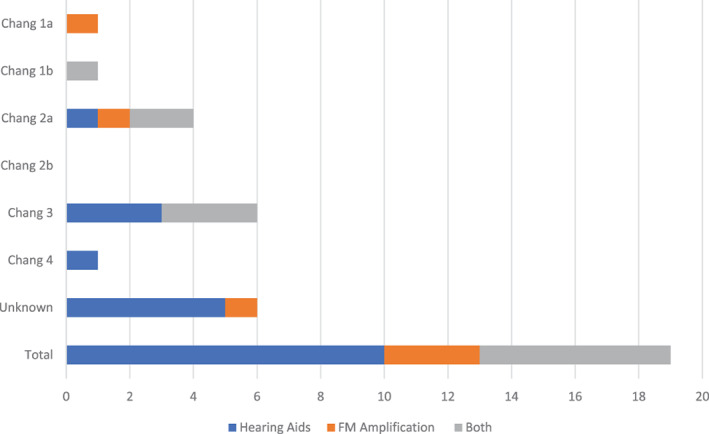
Sub‐analysis of high‐risk (HR) survivors differentiated by Chang grade fitted with either hearing aids, amplifications system, or both. A total of 19 of 26 hearing impaired patients required audiometry support devices. Non‐high risk (NHR) data not shown as only 1 patient was fitted with a hearing aid. FM, frequency modulated

Figure 3 demonstrates sub‐analysis of HR survivors differentiated by Chang grade fitted with either hearing aids, amplification system or both.

### 3.6 Endocrine

**FIGURE 4:**
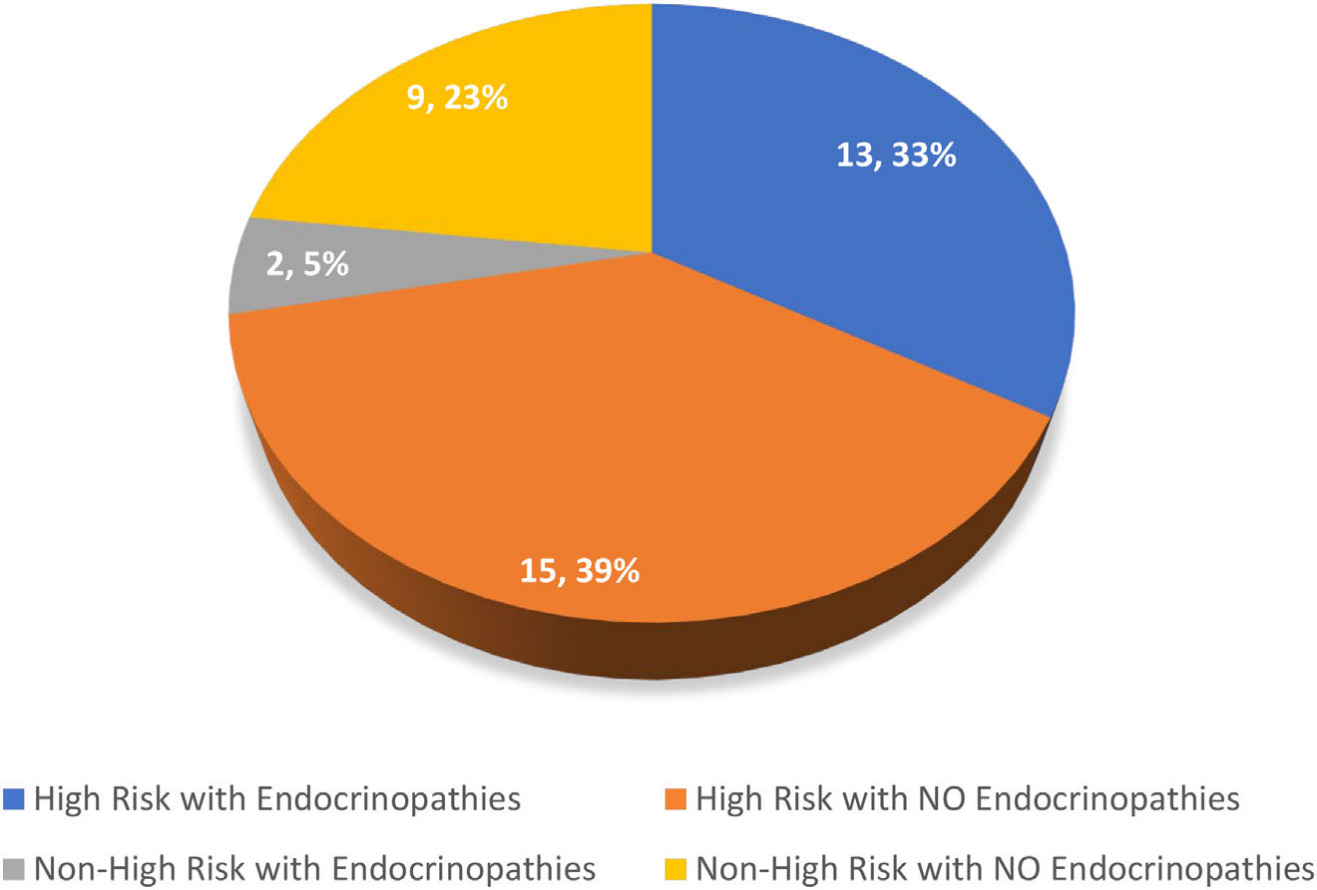
Presence or absence of endocrinopathy by risk group with number and percentage as shown, *n* = 39

Regarding endocrine toxicities, 15/39 (38%) of the full effects patients were found to have some form of endocrinological late effect including bone metabolism disorders (Perthe's disease, avascular necrosis, osteochondromas or osteoporosis), delayed growth, insulin abnormalities, adrenal insufficiency, and thyroid abnormalities. Figure 4 demonstrates the breakdown of the 39 patients with and without endocrinopathies by risk group. There were 28 HR patients and 11 NHR patients making up the total of 39. A total of 13 out of 28 HR patients (46%) suffered an endocrine late effect, versus only 2/11 (18%) of the NHR patients (*p* = .150, FET).

On subset analysis of endocrinopathies, 7/39 patients had stunted growth with 3 patients displaying an advanced bone age, 2 with a *delayed* bone age, 1 requiring growth hormone for significantly impeded height, and 1 for ongoing height surveillance with no active intervention. These children were also found to have other coinciding endocrinopathies including hypothyroidism, gonadal failure requiring supplementation, adrenal insufficiency, fasting cholesterol/triglyceride abnormalities suggesting metabolic syndrome and bone metabolism disorders. Four patients had hypothyroidism requiring treatment with thyroxine. Ten patients were found to have single endocrine issues only, including slow growth velocity with delayed bone age, insulin resistance, hyperinsulinism, osteoporosis, hypothyroidism, a bone metabolism issue as defined above or partial adrenal insufficiency. Endocrinopathies were primarily found among the HR group. Of the 2 NHR patients with endocrinopathies, 1 had hyperinsulinism with risk of Type 2 diabetes mellitus, and the other suffered from osteoporosis.

Among survivors of the late effects cohort (*n* = 27), 13/17 (76%) HR patients had an endocrinopathy in comparison to 1/10 (10%) NHR patients (*p* = .001, FET) totalling 52% (14/27) of survivors with an endocrine late effect.

### 3.7 Cardio‐pulmonary

Of the 39 full late effects patients, 3/39 (8%) suffered a cardiac late effect. The spectrum of cardiac issues included pericarditis with ventricular dysfunction, significant pulmonary hypertension from respiratory failure leading to cardiac failure, and tachyarrhythmias.

Only 37 of the 39 patients were evaluable for pulmonary late effects, as 2 had not received formal testing yet. Five of the 37 (14%) patients were found to have a pulmonary late effect. Pulmonary effects ranged from dyspnea limiting exercise, interstitial lung disease with fibrosis, pulmonary hypertension with eventual type 2 respiratory failure, mild restrictive changes on respiratory testing, to respiratory failure with pulmonary haemorrhage.

### 3.8 Dental

Dental late effects were seen almost exclusively in the HR group. A total of 19 out of 39 (49%) patients analysed demonstrated a variety of dental complications which included a spectrum of short and tapered roots, mixed dentition, microdontia and/or dental underdevelopment. One of 11 NHR patients demonstrated failure of teeth eruption with ectopic teeth, whilst the other 18/28 HR patients demonstrated a spectrum of complications as listed (*p* = .003, FET). Persistence of primary dentition, missing permanent dentition/agenesis of teeth, caries, failure of teeth eruption and ectopic teeth were among other late effects found on exam.

Among survivors of the late effects cohort (*n* = 27) 16/17 (94%) HR patients demonstrated persistent dental late effects in comparison to 0/10 NHR (*p* = <.001, FET). Survivors were all from the HR group as the 1 NHR patient had died secondary to pulmonary toxicity.

### 3.9 Neuro‐cognitive deficits

A total of 46% (18/39) of patients were found to have some form of neurobehavioral impairment among the two risk groups. This domain was heterogenous and included a variety of disorders including attention deficit hyperactivity disorder (ADHD), mood disorders, sleep disorders, language issues, autism spectrum disorder (ASD), speech disorders, academic concerns, learning difficulties, developmental issues, higher cognitive function issues and Tourette's syndrome. When assessing these 18 cases by risk group, 13/28 (46%) were HR and 5/11 (45%) were NHR (*p* = 1.00, FET).

Neurobehavioral impairment was found in 17/27 (63%) survivors in the full effects cohort. There was no difference between the HR and NHR groups (13/17 (76%) versus 4/10 (40%) respectively, *p* = .101, FET).

In sub‐analysis of the 39 patients for learning difficulties specifically, 8/28 (29%) HR patients were found to have learning difficulties versus 3/11 (27%) NHR patients (*p* = 1.00, FET).

Psychological intervention was common in this domain with 16 of the 18 overall affected patients (89%) having received some form of psychological intervention at any time during their follow‐up. Intervention was for neurobehavioral issues such as ASD, ADHD, anxiety, socialization concerns, and learning difficulties. Among survivors of the full effects cohort (*n* = 27), 15/27 (56%) required psychological intervention. There was no difference between the HR and NHR groups in terms of psychology support utilization (11/17 (65%) versus 4/10 (40%), *p* = .257, FET).

### 3.10 Subsequent malignant neoplasm

No SMNs were found on analysis of the 39 late effects patients at follow‐up.

## 4 DISCUSSION

The 5‐year relative survival for neuroblastoma patients has improved from 54% to 81.6%^7^, ^12^ between 1975 and 2018, but as described by Laverdière^7^ and colleagues, long‐term toxicities of treatment in survivors is notable with an 8.3‐fold risk of developing a chronic health condition versus siblings. This balance between improving survival, whilst reducing late effects is challenging, but an increasingly important focus in HR neuroblastoma treatment. In our study, the range of late effects seen were wide but consistent with international cohorts.^2^, ^6^

Our results demonstrated 52% of surviving neuroblastoma patients suffered from an endocrine complication, inclusive of bone metabolism issues, falling within the range of complications seen in the literature.^7^, ^13^, ^14^ Growth effects and hypothyroidism were the most common conditions seen in our cohort, primarily in the HR group suggesting that intense multi‐modality HR therapy has a strong correlation to endocrine late effects.^15^ The effect on growth parameters is most attributable to the use of intensive chemotherapy and 13‐cis‐retinoic acid and its association with premature epiphyseal closure.^15^ The hypothyroidism in our cohort is likely attributed to high use of diagnostic ^131^I‐MIBG despite iodide prophylaxis, and treatment at an early age in most neuroblastoma patients, as suggested by Cohen^15^ and colleagues. Incidence of hypothyroidism is also higher in patients who have been treated with therapeutic ^131^I‐MIBG as the therapeutic agent appears to be linked to thyroid dysfunction.^14^ Pubertal concerns from gonadal failure have also been found in the literature, but perhaps prevalence in our full effects cohort (1/39) was low, as follow‐up had not occurred for a long enough period.^15^ Endocrine late effects are prevalent among neuroblastoma survivors, and so it is imperative that all physicians who manage these patients are aware of such issues.

Dental effects were found almost exclusively in the HR patients, with 94% of survivors having suffered a late effect. This is consistent with data in other cohorts of HR survivors.^16^ The range of dental concerns described above are consistent with findings in the literature and have been linked to ASCT conditioning, vincristine, alkylating agents, and radiotherapy.^6^, ^15^, ^17^ There has also been a dose dependent link described with alkylating agents leading to development of at least one dental effect when compared to survivors with no alkylating agent exposure.^18^ Additionally, poor dental hygiene in survivors has led to commonly found caries on follow‐up with literature evidence to support preventive dental care improving overall dental outcomes.^17^ Treatment of dental issues in adult survivors can also be complicated by long public hospital wait lists, and cost issues in community dental clinics highlighting the importance of preventative care.

Hearing impairment in neuroblastoma survivors has been found to be statistically significant in large cohort studies, especially those treated with HR therapy with cumulative exposure to cisplatin.^7^, ^19^ Mild to moderate high‐frequency loss has significant implications for young children as the high‐frequency sounds required for speech discrimination plays an integral role in academic function.^19^ In our cohort, 93% of HR survivors were hearing impaired with most of our HR survivors requiring either hearing aids or an FMAS, but only 14 patients received additional platinum therapy during conditioning, suggesting that intensive use of cisplatin in induction contributes significantly to ototoxicity. Hearing loss impacts multiple outcomes including speech and language development, psychosocial development, and academic performance.^20^ Moreover, the sensorineural hearing loss associated with cisplatin has been suggested to be progressive,^2^, ^21^ so regular screening is imperative to manage morbidity and impacts on future quality of life.

Induction therapy in both the Society of Pediatric Oncology European Neuroblastoma Group (SIOPEN) and COG trials are heavily platinum‐based, with carboplatin also being used in COG consolidation phases.^5^ Given these platinum‐based backbones, risk of ototoxicity is high necessitating the need for stringent screening and early intervention to prevent delays in language and psychosocial development. Additionally, as event free survival (EFS) has been shown to be better in tandem versus single transplant in HR neuroblastoma, the difficulty in reducing platinum‐based therapy is compounded when carboplatin is used as part of conditioning.^22^

Otoprotection has been investigated in various trials with sodium thiosulfate (STS) showing some promise of otoprotection, without compromising EFS, or overall survival (OS) in a multi‐centre open‐label randomized trial (SIOPEL 6).^23^ These results were however contradicted by another multi‐centre, open‐label randomized trial (ACCL0431) with a more heterogenous group of patients suggesting that STS administration negatively impacted the EFS and OS.^24^ Currently, comprehensive trials that accurately assess otoprotection and disease control in children receiving cisplatin therapy are lacking, requiring further analysis through studies limited to children with the same malignancy type, treatment regime and response evaluation.^25^

Neuro‐cognitive deficits have also been evident in neuroblastoma survivors, with 46% of our full effects cohort displaying some form of neuro‐cognitive deficit. A total of 56% of survivors received some form of psychological intervention during follow‐up, suggesting psychological support is an important and significant factor in late effects management. In a study by Zheng^26^ and colleagues, neuroblastoma survivors who had two or more chronic issues were more likely to have impairment in psychosocial domains. They also found that compared to siblings, survivors were at greater risk of worse outcomes in academic achievement and were more likely to require educational support. This is further supported by evidence that neuroblastoma survivors have lower emotional wellbeing.^27^ Studies in adult survivors also found they were less likely to be employed^7^ or married and had lower individual and household incomes versus siblings. Overall, psychosocial outcomes are a significant factor in survivor health and social integration, demonstrating the importance of early intervention and screening.

A total of 72% of our full cohort was alive at follow‐up and 64% of survivors were disease free suggesting improved survival with contemporary treatment as reported by other international studies.^26^ Survivors are now being diagnosed with chronic medical conditions at an alarming rate with the 20‐year cumulative incidence of overall chronic health conditions at 41%.^7^

Potential ways to ameliorate the chronic health conditions in survivors include improved risk stratification and tailoring of therapy‐based on response so that intensive therapy is used in poor responders potentially sparing good responders from treatment toxicity. Risk stratification varies among different cooperative groups but as the “omic” technologies including genomics, transcriptomics and proteomics improve, identification of biomarkers of response will greatly improve allowing for more accurate risk categorization and tailoring of therapy. Current risk stratification such as the International Neuroblastoma Risk Group (INRG) classification was designed as a standardized system that combines multiple prognostic factors to define pre‐treatment risk groups, but was unable to incorporate “omic” technologies at the time of guideline creation and so future versions are anticipated to incorporate molecular techniques allowing for more accurate prognostication.^28^ The incorporation of molecularly targeted, and other novel therapies including ^131^I‐MIBG with radiosensitizers, and immunotherapy may also support the possibility of a wider therapeutic window with a decreased toxicity profile.^29^

Additionally, liquid biopsy studies have demonstrated that cell‐free DNA (cfDNA) is a sensitive method of detecting minimal residual disease (MRD) in adult cancers and has been used for risk stratification in adult clinical trials.^30^ Liquid biopsy could likewise be applied in neuroblastoma patients,^31^ where cfDNA could be used to guide intensification or de‐intensification of treatment as children progress through therapy, with the aim to reduce the burden of late effects if a good MRD response is obtained. It is important to emphasize that current risk stratification and treatment strategies need to be improved to reduce the significant burden of late effects to allow for improved patient management, with the hope that modern MRD techniques and molecularly targeted agents will inform future customized therapies.

Limitation of this study included a small cohort of 39 being analysed for full effects, as only these patients had reached 5 years off treatment. Another limitation is the duration of follow‐up, where only a small proportion of the 39 cases would have reached 10 years of follow‐up, which would not have been long enough to assess for all late effects, especially SMN, cardiometabolic and endocrine late effects. For instance, there were no SMN in our cohort, but this was likely because follow‐up had not occurred for long enough. Additionally, this is a single institution study based on retrospective record review so only details clearly documented in medical records were analysed.

This study highlights that the burden of chronic disease in neuroblastoma survivors is significant and ongoing multidisciplinary management is required for prompt diagnosis and intervention.

## AUTHOR CONTRIBUTIONS

**Veronica Yeung:** Conceptualization (equal); data curation (lead); formal analysis (lead); project administration (lead); writing – original draft (lead); writing – review and editing (lead). **Melissa Gabriel:** Conceptualization (equal); methodology (equal); supervision (supporting); writing – review and editing (equal). **Bhavna Padhye:** Conceptualization (equal); methodology (equal); resources (lead); supervision (lead); writing – review and editing (equal).

## CONFLICT OF INTEREST

The authors have stated explicitly that there are no conflicts of interest in connection with this article.

## ETHICS STATEMENT

This study has been completed in accordance with the ethical guidelines as published on the Wiley author services on Research Integrity and Publishing Ethics. It has been performed in an ethical and responsible manner with no research misconduct. Ethics approval was obtained through the Human Research Ethics Committee (HREC) under the Sydney Children's Hospital Network and operates in accordance with the National Health and Medical Research Council's National Statement on Ethical Conduct in Human Research and CPMP/ICH Note for Guidance on Good Clinical Practice; Reference number 2019/ETH11899. Patient consent was not required for this study as this was a retrospective review of medical records with no contact with patients, parents/guardians, or family members; a waiver of consent was approved.

## Supporting information


**Table S1** Chang grades of hearing impairment.

**Table S2** Common terminology criteria for adverse events (CTCAE) v5.0 grades of hearing impairment from the Ear and Labyrinth Disorders section.

**Table S3** Breakdown of grades of audiometric impairment in survivors (*n* = 28).

## Data Availability

The data that support the findings of this study are available on request from the corresponding author. The data are not publicly available due to privacy or ethical restrictions.
