# Supplementary material for: Late effects and treatment related morbidity associated with treatment of neuroblastoma patients in a tertiary paediatric centre
Source: Cancer Rep (Hoboken). 2022 Oct 21;6(3):e1738. doi: 10.1002/cnr2.1738 (PMC10026287; doi:10.1002/cnr2.1738)
Supplement: Supplementary file 1 — Table S1 Chang grades of hearing impairment. [file CNR2-6-e1738-s001.docx]

**Supplemental Table S1** Chang grades of hearing impairment^b^

| Chang Grade Sensorineural Hearing Threshold (dB) |
| --- |
| 0 ≤ 20 dB at 1, 2, and 4 kHz |
| 1a ≥ 40 dB at any frequency 6 to 12 kHz |
| 1b > 20 and < 40 dB at 4 kHz |
| 2a ≥ 40 dB at 4 kHz and above |
| 2b > 20 and < 40 dB at any frequency below 4 kHz |
| 3 ≥ 40 dB at 2 or 3 kHz and above |
| 4 ≥ 40 dB at 1 kHz and above |

^b^Adapted from “Practical grading system for evaluating cisplatin ototoxicity in children”^10(p3)^
