# Supplementary material for: Late effects and treatment related morbidity associated with treatment of neuroblastoma patients in a tertiary paediatric centre
Source: Cancer Rep (Hoboken). 2022 Oct 21;6(3):e1738. doi: 10.1002/cnr2.1738 (PMC10026287; doi:10.1002/cnr2.1738)
Supplement: Supplementary file 2 — Table S2 Common terminology criteria for adverse events (CTCAE) v5.0 grades of hearing impairment from the Ear and Labyrinth Disorders section. [file CNR2-6-e1738-s003.docx]

**Supplemental Table S2** CTCAE v5.0 grades of hearing impairment from the Ear and Labyrinth Disorders section^a^

| CTCAE Term | Grade 1 | Grade 2 | Grade 3 | Grade 4 | Grade 5 |
| --- | --- | --- | --- | --- | --- |
| Hearing Impairment | Pediatric (on a 1,2,3,4,6, and 8 kHz audiogram): Threshold shift >20 dB hearing loss (HL) (i.e., 25 dB HL or greater); sensorineural hearing loss (SNHL) above 4 kHz (i.e., 6 or 8 kHz) in at least one ear | Pediatric (on a 1, 2, 3, 4, 6, and 8 kHz audiogram): Threshold shift >20 dB at 4 kHz in at least one ear | Pediatric (on a 1, 2, 3, 4, 6, and 8 kHz audiogram): Hearing loss sufficient to indicate therapeutic intervention, including hearing aids; threshold shift >20 dB at 2 to < 4 kHz in at least one ear | Pediatric: Audiologic indication for cochlear implant; > 40 dB HL (i.e., 45 dB HL or more); SNHL at 2 kHz and above | N/A |

^a^Adapted from “CTCAE v5.0 Nov. 27, 2017”^11(p13)^
Abbreviations: CTCAE, common terminology criteria for adverse events; N/A, not applicable
