# Supplementary material for: Late effects and treatment related morbidity associated with treatment of neuroblastoma patients in a tertiary paediatric centre
Source: Cancer Rep (Hoboken). 2022 Oct 21;6(3):e1738. doi: 10.1002/cnr2.1738 (PMC10026287; doi:10.1002/cnr2.1738)
Supplement: Supplementary file 3 — Table S3 Breakdown of grades of audiometric impairment in survivors (n = 28). [file CNR2-6-e1738-s002.docx]

**Supplemental Table S3** Breakdown of grades of audiometric impairment in survivors
(n =28)

| Grade of Audiometric Impairment | High Risk Total  26 (out of 28 survivors) | Non-High-Risk Total  2 (out of 19 survivors) | Overall Total  28 (out of 47 survivors) |
| --- | --- | --- | --- |
| Chang 1a | 6 | 0 | 6 |
| Chang 1b | 2 | 0 | 2 |
| Chang 2a | 5 | 0 | 5 |
| Chang 3 | 6 | 0 | 6 |
| Chang 4 | 1 | 0 | 1 |
| Unknown | 6 | 2 | 8 |
